# Supplementary material for: Systematic multi-trait AAV capsid engineering for efficient gene delivery
Source: Nat Commun. 2024 Aug 4;15:6602. doi: 10.1038/s41467-024-50555-y (PMC11297966; doi:10.1038/s41467-024-50555-y)
Supplement: Supplementary file 1 — Supplementary Information [file 41467_2024_50555_MOESM1_ESM.pdf]

Supplementary Information for

# Systematic multi-trait AAV capsid engineering for efficient gene delivery

Fatma-Elzahraa Eid*, Albert T. Chen, Ken Y. Chan, Qin Huang, Qingxia Zheng, Isabelle G. Tobey, Simon Pacouret, Pamela P. Brauer, Casey Keyes, Megan Powell, Jencilin Johnston, Binhui Zhao, Kasper Lage, Alice F. Tarantal, Yujia A. Chan, Benjamin E. Deverman*

Correspondence to: fatma@broadinstitute.org and bdeverma@broadinstitute.org

**Contents:**

Supplementary Figures 1 to 9

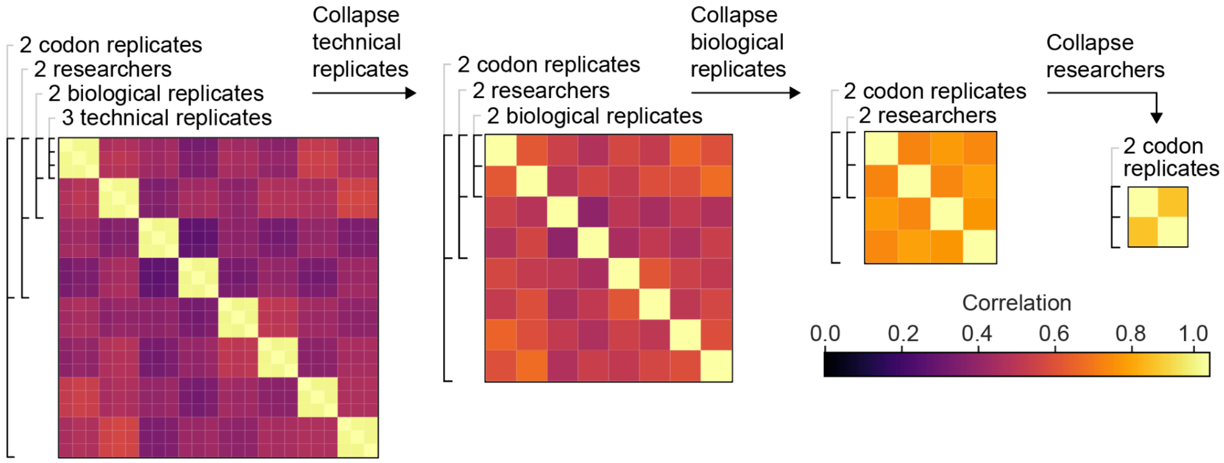


**Supplementary Fig. 1. Production fitness replication quality improves upon hierarchical aggregation of replicates.** The replication quality between replicates was assessed by the Pearson correlation of $\log_2$ reads per million (RPM) between replicates. The replication quality is shown for data that was aggregated (averaged) by technical replicates, then biological replicates, and then by researchers.

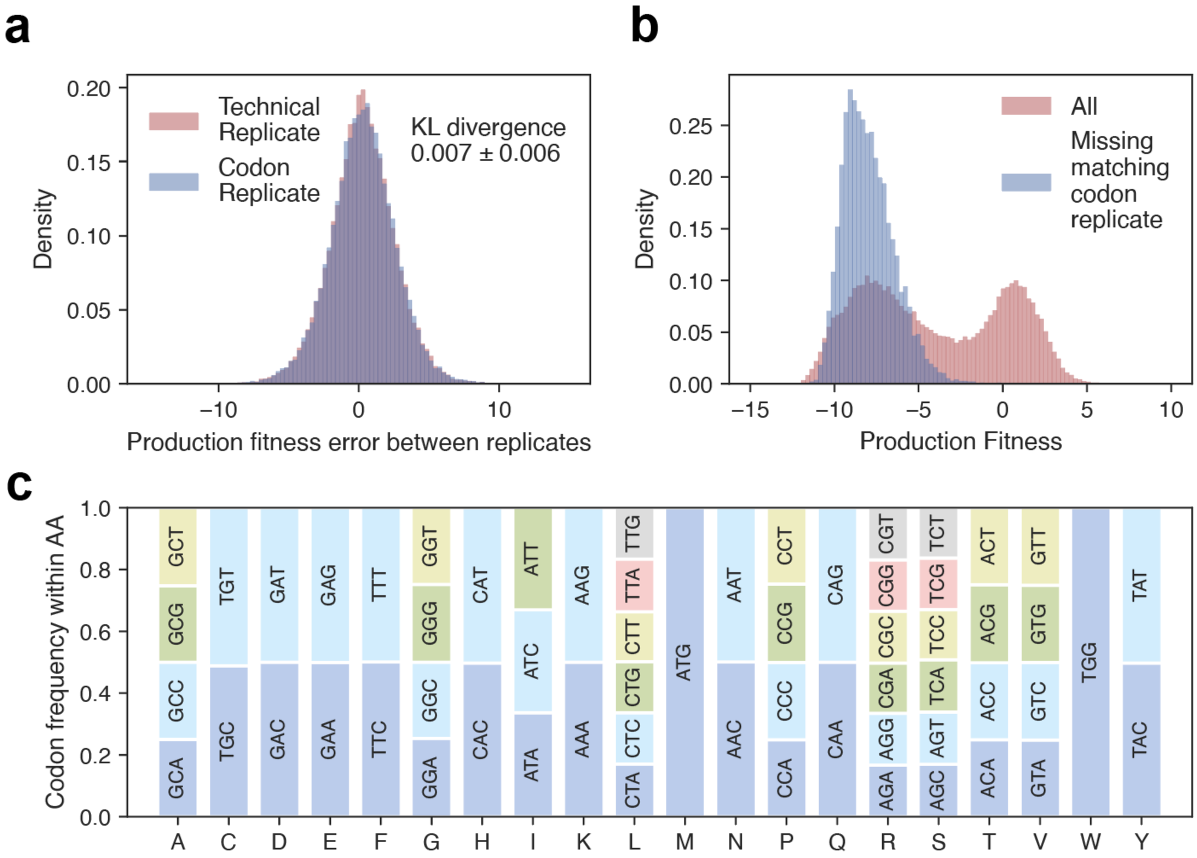


**Supplementary Fig. 2. Codon usage of 7-mer insertions minimally affects capsid production fitness.** (**a**) The distribution of the difference in production fitness scores measured between codon replicate pairs and between technical replicate pairs are similar (Kullback-Leibler divergence = 0.006±0.007). (**b**) The variants with a single codon replicate detected (missing matching codon replicate) had production fitness scores on the low end of the production fitness bimodal distribution. Of the 13,217 7-mer sequences where only one of the two codon replicates was detected in the AAV library (20.5% of the 64,500 amino acid variants), >99% had fitness scores on the low end of the fitness distribution, suggesting that the missing replicates were not detected due to low abundance. (**c**) The codon usage distribution in the modeling library follows the expected uniform distribution for each amino acid.

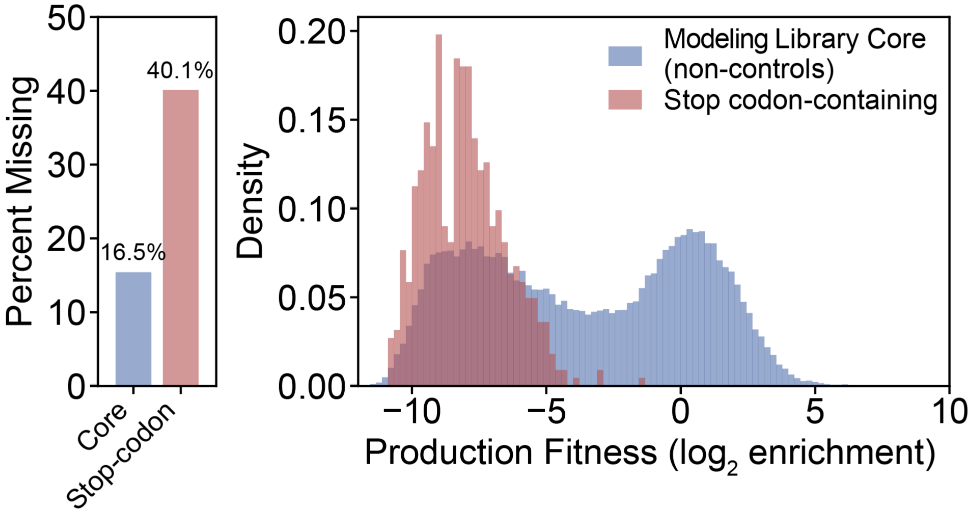


**Supplementary Fig. 3. Distinguishing production-fit and non-fit distributions.** In the AAV library, 40.1% of the stop codon-containing sequences were not detected as compared to 16.5% of the sequences without stop codons. The detected stop-codon-containing variants in the modeling library, presumably arising due to cross-packaging, overlapped with the non-fit distribution in the production fitness landscape (codon replicates were not aggregated).

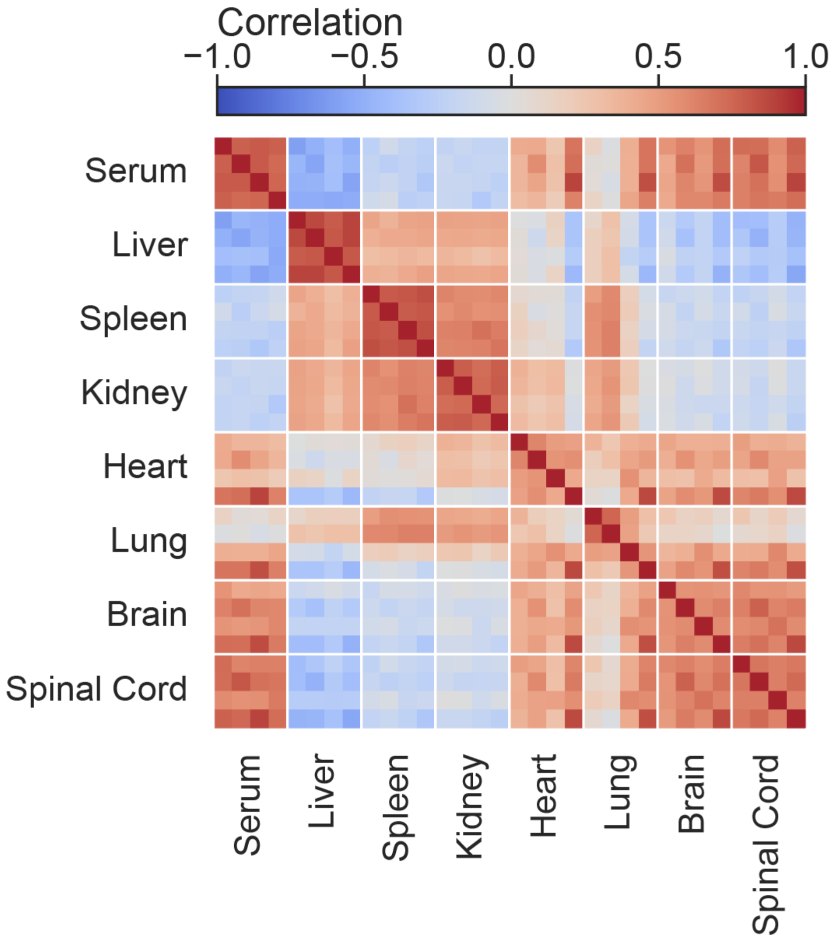


**Supplementary Fig. 4. Fit4Function variant biodistribution correlation between organs.** The pairwise Pearson correlation of organ biodistribution fitness ($\log_2$ enrichment scores) between C57BL/6J mice (*n* = 4 animals) are shown.

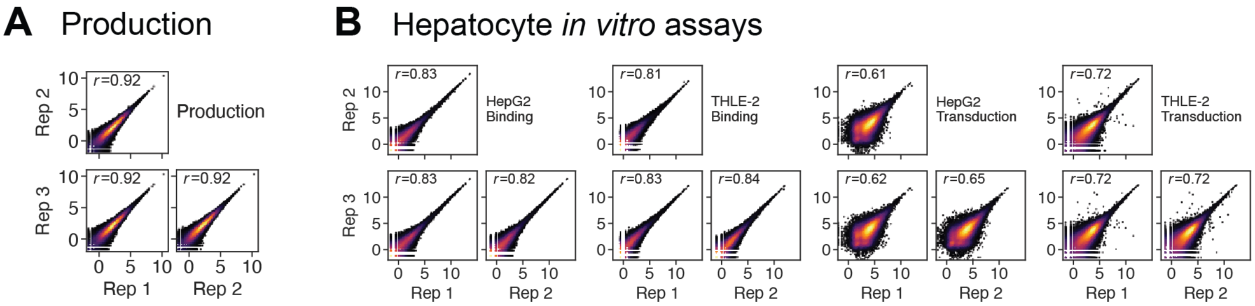


**Supplementary Fig. 5. The replicability of the *in vitro* assays used to screen the Fit4Function library.** The pairwise correlations between biological triplicates are shown for (**a**) production fitness and (**b**) HEPG2 binding or transduction and THLE-2 binding or transduction. The replicate numbers (Rep) are arbitrary and are not linked across assays.

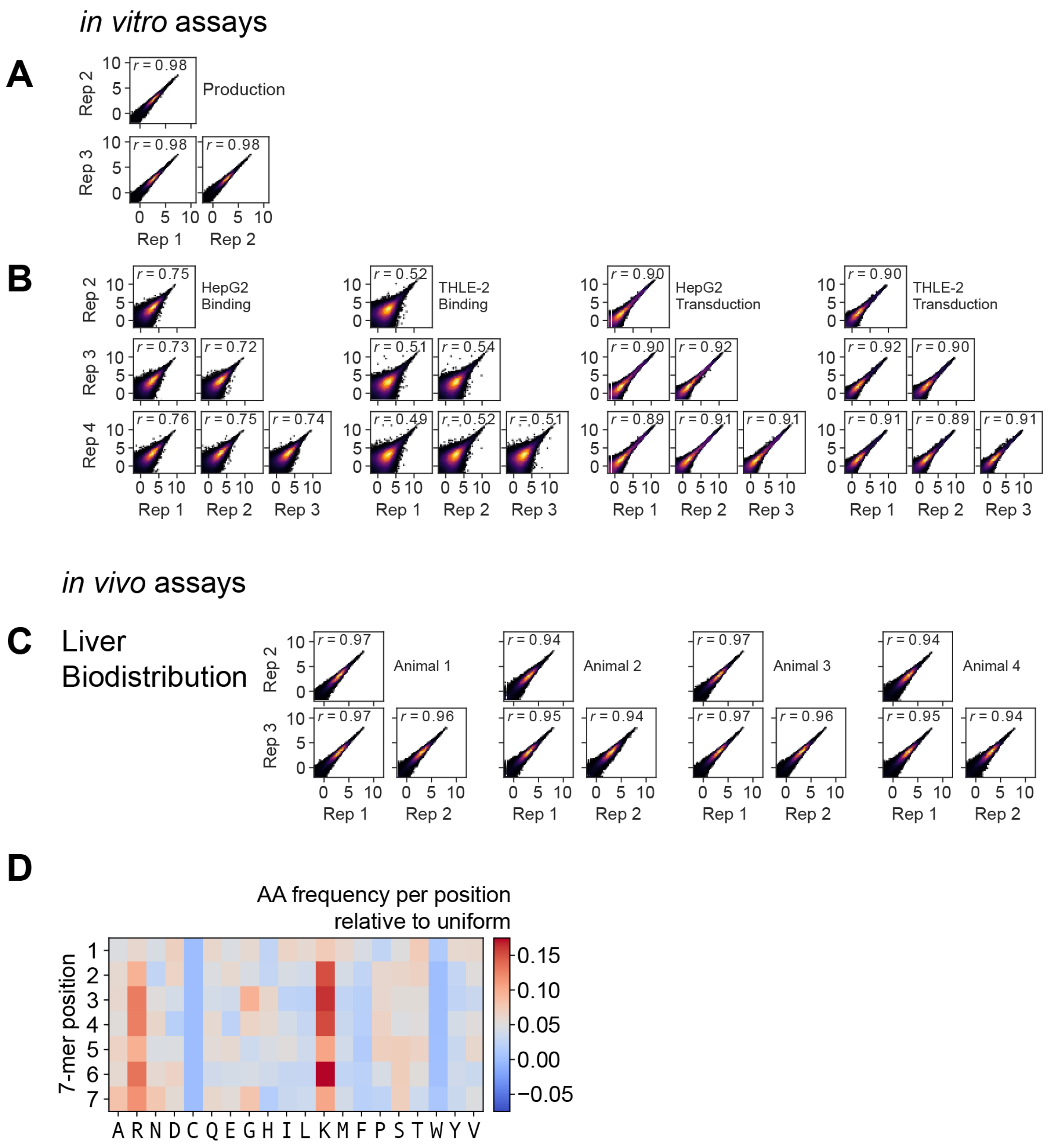


**Supplementary Fig. 6. The replicability of the MultiFunction library across *in vitro* and *in vivo* assays.** The pairwise correlations between biological triplicates are shown for (**a**) production fitness, (**b**) *in vitro* human cell binding and transduction, and (**c**) *in vivo* liver biodistribution in C57BL/6J mice. The replicate numbers (Rep) are arbitrary and are not linked across assays. (**d**) The amino acid distribution by position is shown for the variants in the liver MultiFunction set.

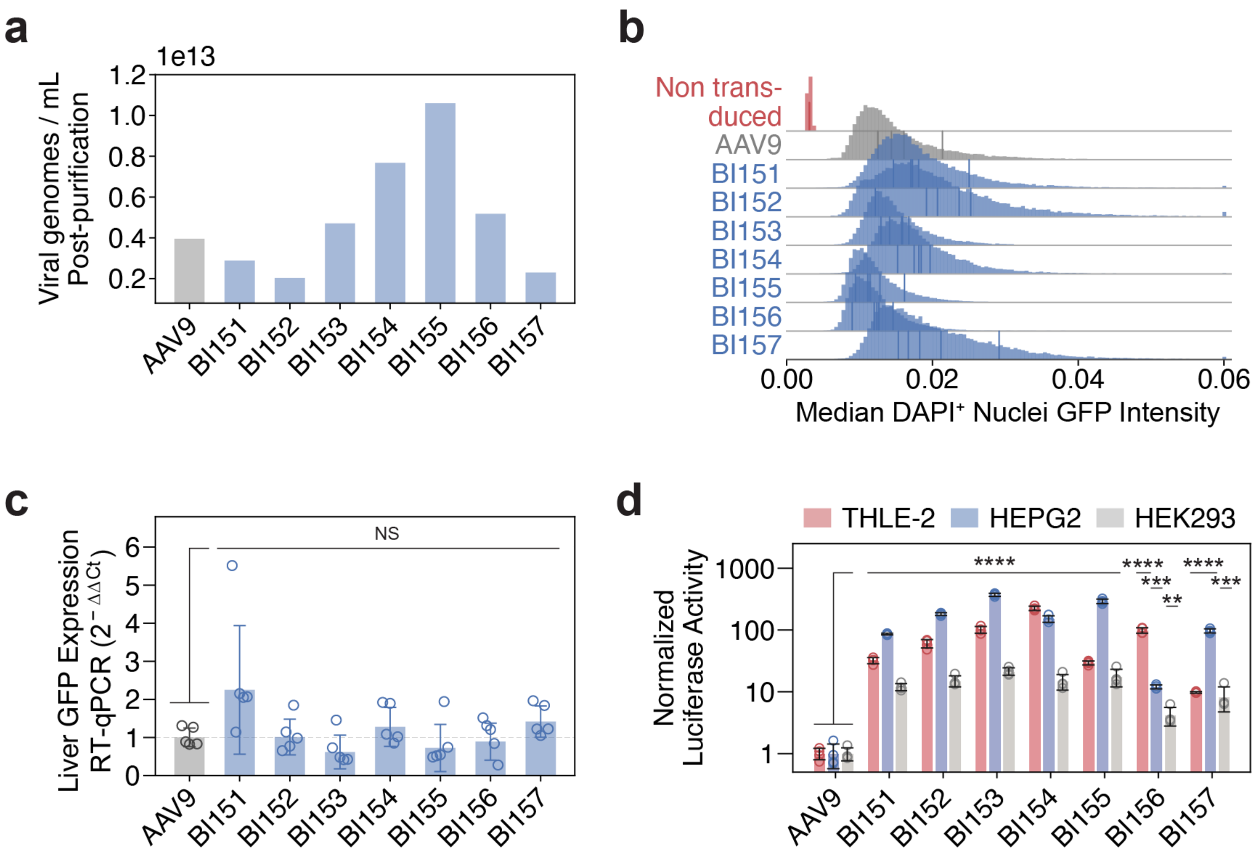


**Supplementary Fig. 7. Individual assessment of liver MultiFunction capsids for production and cell transduction.** (**a**) The production yields from 200 mL of cell culture are shown for each of the individually manufactured capsids. (**b**) C57BL/6J liver transduction by AAV9 or MultiFunction capsids are shown. Mice were injected with $1x10^{10}$ vg of the indicated capsid packaging AAV-CAG-GFP-2A-Luc-WPRE-pA and assessed for GFP expression three weeks later (*n* = 5 mice for each AAV treatment condition, *n* = 3 mice for the no AAV control, all BI capsids were not significantly different from AAV9 in unpaired, one-sided t-tests with Bonferroni correction). The distributions of the median GFP pixel intensity per $DAPI^+$ nuclei, combined across *n* = 5 animals for each AAV treatment condition or *n* = 3 animals for the no AAV control are shown. The vertical lines within each distribution represent the mean of each animal. (**c**) AAV9 or the indicated variant was systemically administered to C57BL/6J mice at $1x10^{10}$ vg/mouse. Three weeks later, liver transduction was measured by RT-qPCR of AAV transcripts from extracted tissue. The ΔΔCt was obtained by normalizing against the reference gene (GAPDH), and then against the AAV9 control (*n* = 5 animals/group; mean ± s.d., unpaired, one-sided t-tests on log-transformed values, and Bonferroni corrected for multiple hypotheses). (**d**) Human liver cell line (THLE-2, HEPG2) and HEK293 transduction 24 hours after exposure to 5000 vg/cell of each indicated capsid packaging AAV-CAG-GFP-2A-Luc (*n* = 4 per group, mean ± s.d., **$p$<0.01, ***$p$<0.001, ****$p$<1e-4 unpaired one-sided t-tests on log-transformed values, and Bonferroni correction for multiple hypotheses) are shown as normalized to AAV9.

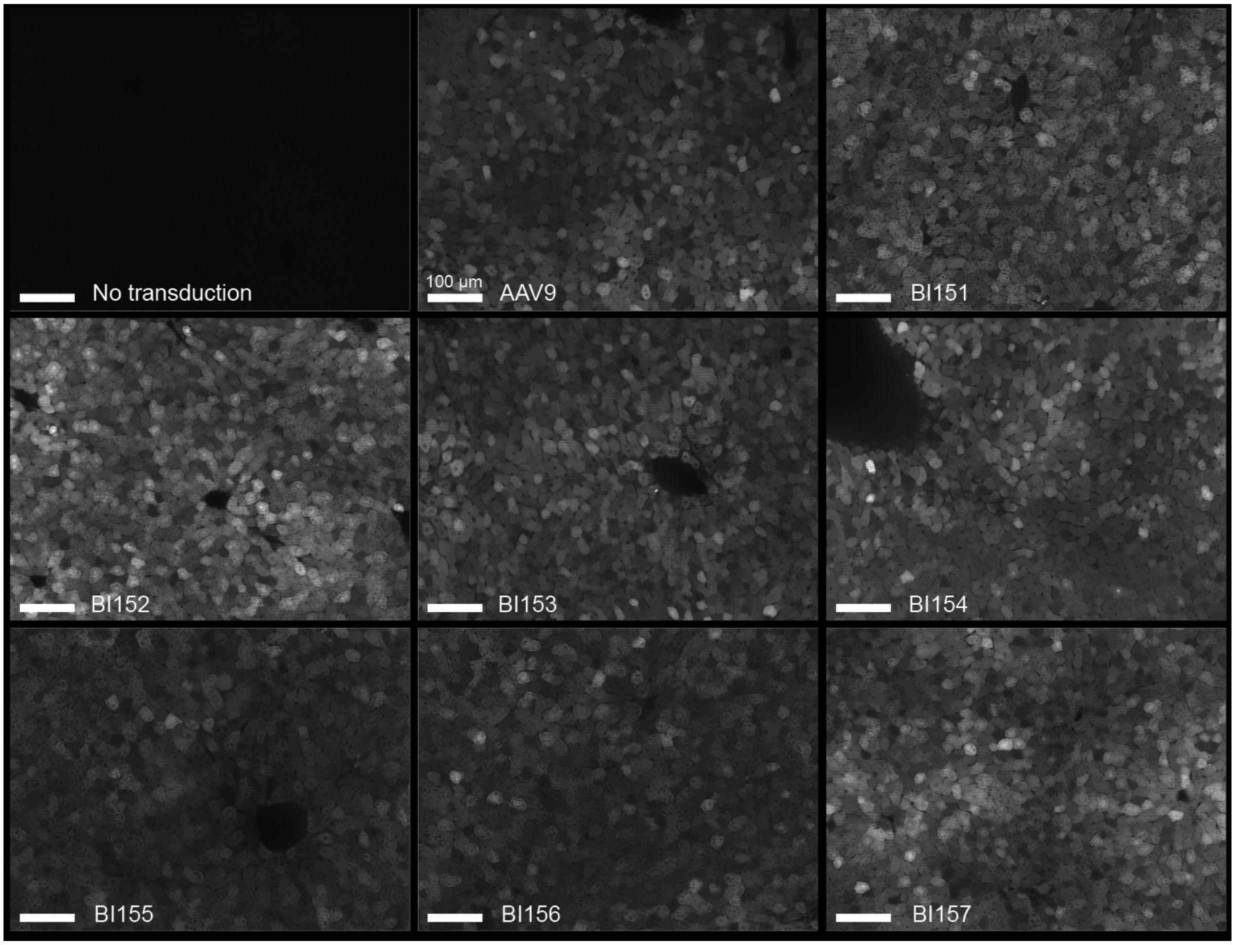


**Supplementary Fig. 8. *In vivo* mouse liver transduction by each MultiFunction capsid.** The 6 to 8-week-old C57BL/6J mice were injected with $1x10^{10}$ vg of each AAV and harvested at 3 weeks post-injection. Representative GFP images of liver slices are shown for the no AAV control, AAV9, and BI variants. Images were chosen from the median replicate of the median animal per condition. All images were taken at the same exposure and rescaled to the same intensity range. The scale bar in all images = 100 µm. The images shown are representative of data collected from five animals per group. The full quantitative analysis of expression from all replicates in this experiment is shown in Supplementary Fig. 7b.

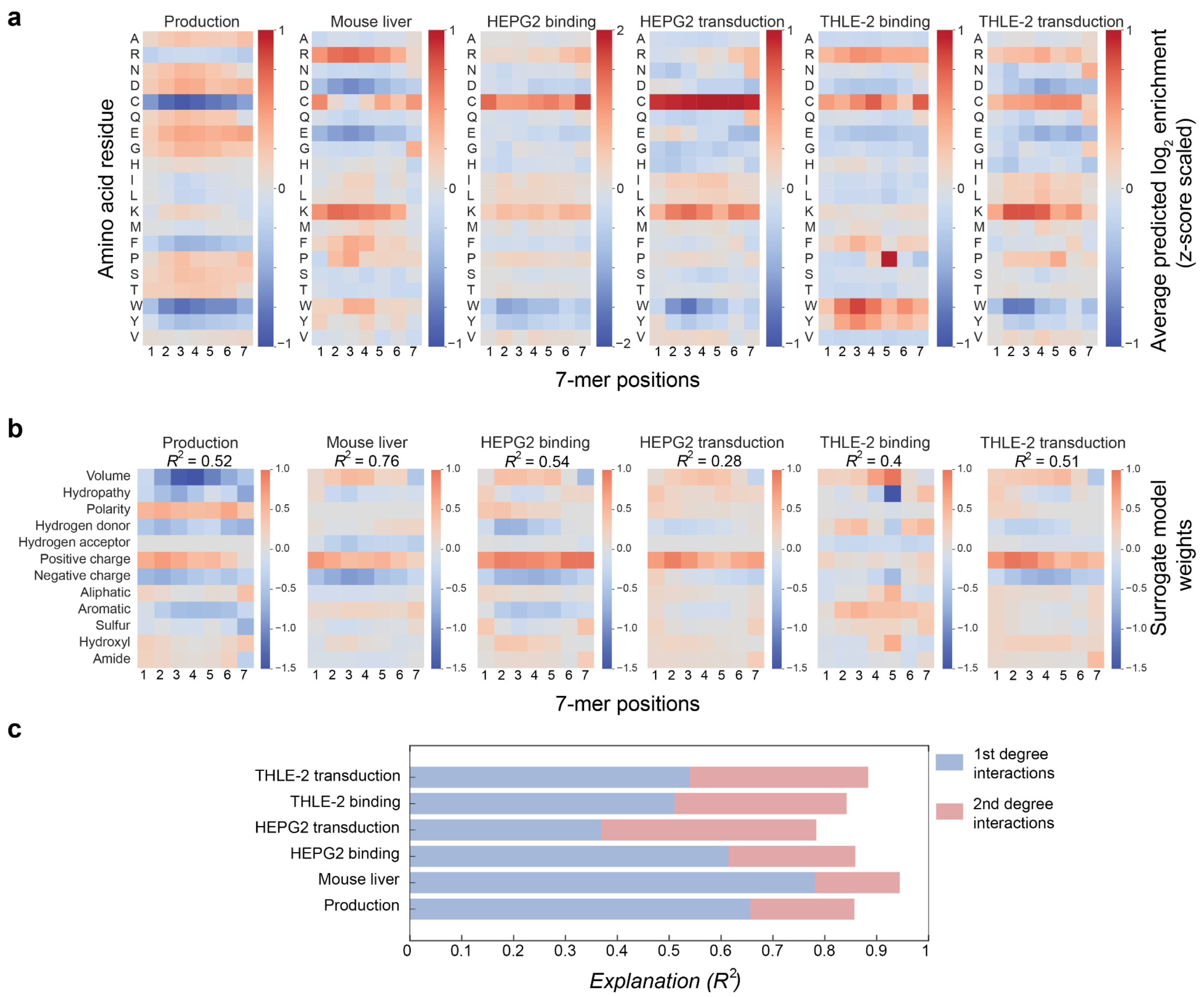


**Supplementary Fig. 9. Interpreting the production fitness and liver-related functional fitness models.** (**a**) Each cell in the heatmap represents the average predicted $\log_2$ enrichment when the indicated amino acid residue is held fixed at the indicated position in the 7-mer while the residues at other positions are randomized. Values are z-score normalized. (**b**) Surrogate concept linear regression lasso models were trained on the indicated physicochemical properties of the amino acid residues in each position as opposed to the amino acid residue itself. Each cell represents the weight of each indicated property at each position pertaining to the predicted function; positive or negative weights indicate contribution, positively or negatively, to the predicted functional score. The agreement ($R^2$) between the predictions of each original model and those of the surrogate models are shown; a low $R^2$ agreement indicates that the signal driving the surrogate model predictions cannot fully account for the original model's predictions. (**c**) The agreement ($R^2$) between the predictions of each original model and those of the surrogate models trained on first-degree interactions only or both first- and second-degree interactions are shown.
