# Supplementary material for: Systematic multi-trait AAV capsid engineering for efficient gene delivery
Source: Nat Commun. 2024 Aug 4;15:6602. doi: 10.1038/s41467-024-50555-y (PMC11297966; doi:10.1038/s41467-024-50555-y)
Supplement: Supplementary file 2 — Reporting Summary [file 41467_2024_50555_MOESM2_ESM.pdf]

nature portfolio

Corresponding author(s): Benjamin E. Deverman and Fatma Elzahraa Eid

Last updated by author(s): 10/02/2023

Reporting Summary

Nature Portfolio wishes to improve the reproducibility of the work that we publish. This form provides structure for consistency and transparency in reporting. For further information on Nature Portfolio policies, see our Editorial Policies and the Editorial Policy Checklist.

Statistics

For all statistical analyses, confirm that the following items are present in the figure legend, table legend, main text, or Methods section.

n/a	Confirmed	
☐	☒	The exact sample size (*n*) for each experimental group/condition, given as a discrete number and unit of measurement
☐	☒	A statement on whether measurements were taken from distinct samples or whether the same sample was measured repeatedly
☐	☒	The statistical test(s) used AND whether they are one- or two-sided<br>*Only common tests should be described solely by name; describe more complex techniques in the Methods section.*
☐	☒	A description of all covariates tested
☐	☒	A description of any assumptions or corrections, such as tests of normality and adjustment for multiple comparisons
☐	☒	A full description of the statistical parameters including central tendency (e.g. means) or other basic estimates (e.g. regression coefficient) AND variation (e.g. standard deviation) or associated estimates of uncertainty (e.g. confidence intervals)
☐	☒	For null hypothesis testing, the test statistic (e.g. *F*, *t*, *r*) with confidence intervals, effect sizes, degrees of freedom and *P* value noted<br>*Give P values as exact values whenever suitable.*
☒	☐	For Bayesian analysis, information on the choice of priors and Markov chain Monte Carlo settings
☒	☐	For hierarchical and complex designs, identification of the appropriate level for tests and full reporting of outcomes
☐	☒	Estimates of effect sizes (e.g. Cohen's *d*, Pearson's *r*), indicating how they were calculated

Our web collection on statistics for biologists contains articles on many of the points above.

Software and code

Policy information about availability of computer code

Data collection	We used bcl2fastq (version v2.20.0.422) for NGS data de-multiplexing. Alignment was performed with bowtie2 (version 2.4.1). Resulting sam files from bowtie2 were sorted by read and compressed to bam files with samtools (version 1.11-2-g26d7c73, htslib version 1.11-9-g2264113). Python (version 3.8.3) scripts and pysam (version 0.15.4) were used to extract the 21 nucleotide insertion from each amplicon read. Python (version 3.8.3) scripts were used for preprocessing the data as described in details in the Methods.
Data analysis	Python 3 was used to write custom code for the visualization, analyses and modeling of the data in the study. All data, material, and algorithms are described in the main text or supplementary materials. Code and associated experimental data produced in the study that are needed to reproduce, verify, and extend the study are available in the Zenodo repository under accession code 10.5281/zenodo.8401253. Updated code will be maintained in the GitHub repository: https://github.com/vector-engineering/Fit4Function

For manuscripts utilizing custom algorithms or software that are central to the research but not yet described in published literature, software must be made available to editors and reviewers. We strongly encourage code deposition in a community repository (e.g. GitHub). See the Nature Portfolio guidelines for submitting code & software for further information.

Data

Policy information about availability of data

All manuscripts must include a data availability statement. This statement should provide the following information, where applicable:

- Accession codes, unique identifiers, or web links for publicly available datasets
- A description of any restrictions on data availability
- For clinical datasets or third party data, please ensure that the statement adheres to our policy

Data generated in the study that are needed to reproduce, verify, and extend the findings of the study are available in a Zenodo repository under accession code https://doi.org/10.5281/zenodo.8401253. Source data files required for reproducing the manuscript plots are provided in the Zenodo repository https://doi.org/10.5281/zenodo.8388031. as 'Source Data Files.zip'. In addition, NGS data will be made available in the NCBI Sequence Read Archive at the time of publication under BioProject ID: PRJNA1131359.

Human research participants

Policy information about studies involving human research participants and Sex and Gender in Research.

Reporting on sex and gender	N/A
Population characteristics	N/A
Recruitment	N/A
Ethics oversight	N/A

Note that full information on the approval of the study protocol must also be provided in the manuscript.

Field-specific reporting

Please select the one below that is the best fit for your research. If you are not sure, read the appropriate sections before making your selection.

[x] Life sciences [ ] Behavioural & social sciences [ ] Ecological, evolutionary & environmental sciences

For a reference copy of the document with all sections, see nature.com/documents/nr-reporting-summary-flat.pdf

Life sciences study design

All studies must disclose on these points even when the disclosure is negative.

Sample size	No sample size calculations were performed prior to experimentation. Sample sizes for library screens (n=3-4) and individual capsid characterization (n=5) were set based on prior experience (Deverman et al Nature Biotechnology 2016; Chen et al Nature Neuroscience 2017; Kumar et al Nature Methods 2020).
Data exclusions	No animals or samples were excluded from the analysis. The variant BI152 was excluded from the rhesus macaque study due to a library assembly error.
Replication	The reproducibility of the production fitness scores was tested via 10K variants common to the assessment library and modeling library as shown in Fig. 2d. Biological triplicates were performed for the binding or transduction of different cell lines using the Fit4Function library versus an NNK library and their reproducibility was quantified via pairwise Pearson correlation as shown in Fig. 3c and Supplementary Fig. 5. The Fit4Function library was screened in four mice, and the reproducibility of the biodistribution in eight organs was assessed in the form of replication quality between pairs of animals as shown in Fig. 3e and Supplementary Fig. 4. The MultiFunction library was screened across in vitro (three replicates for production fitness, four replicates for binding and transduction) and in vivo (n = 3 mice) assays, and the reproducibility within each assay was assessed in the form of replication quality between pairs of replicates as shown in Supplementary Fig. 6. For the comparison between AAV9 and the seven MultiFunction variants, in vitro cell transduction was assessed with four replicates per group (Fig. 4e and Supplementary Fig. 7d; error bars show the standard deviation from the mean) and unpaired, one-sided t-tests were conducted on log-transformed values, with Bonferroni correction for multiple-hypotheses. The in vivo characterization was performed using five female mice per group except for the no injection control group (Supplementary Fig. 7b and c, n = 3 mice; error bars in Supplementary Fig. 7c show the standard deviation from the mean), and unpaired, one-sided t-tests were conducted on log-transformed values, with Bonferroni correction for multiple-hypotheses. Other statistical tests are described in the text. The number of replicates was chosen based on prior data that indicated large effect sizes. ML models were trained in repetition with randomized subsampling and parameterization to ensure robustness. Training and testing sample sizes are described in the Methods section for each model. Models were tested using independent (blind) datasets where possible, i.e., testing was conducted using an independent assessment library for production fitness (Fig. 2f) and independent measurements in a separate animal (Fig. 3f).
Randomization	Mice were randomly assigned to groups based on predetermined sample sizes.

Blinding	Experimenters were not blinded to the sample groups since the data were generated not manually but by NGS or cell counting software for example, which makes it less likely for the experimenter's bias to influence the results. Models were tested using independent (blind) datasets where possible, i.e., testing was conducted using an independent assessment library for production fitness (Fig. 2f) and independent measurements in a separate animal (Fig. 3f).

Reporting for specific materials, systems and methods

We require information from authors about some types of materials, experimental systems and methods used in many studies. Here, indicate whether each material, system or method listed is relevant to your study. If you are not sure if a list item applies to your research, read the appropriate section before selecting a response.

Materials & experimental systems

n/a	Involved in the study
☒	☐ Antibodies
☐	☒ Eukaryotic cell lines
☒	☐ Palaeontology and archaeology
☐	☒ Animals and other organisms
☒	☐ Clinical data
☒	☐ Dual use research of concern

Methods

n/a	Involved in the study
☒	☐ ChIP-seq
☒	☐ Flow cytometry
☒	☐ MRI-based neuroimaging

Eukaryotic cell lines

Policy information about cell lines and Sex and Gender in Research

Cell line source(s)	The following cell lines were used: HEK293T/17 (ATCC® CRL-11268™), HepG2 (ATCC® HB-8065™), THLE-2 (ATCC® CRL-2706™), hCMEC/D3 (Millipore, SCC066), and human and mouse BMVECs (Cell Biologics, H-6023 and C57-H6023).
Authentication	Among the listed cell lines, HEK293T/17 is the only cell line known to be potentially cross-contaminated with HeLa cells. We used morphology checks under light microscopy with a Leica DM IL LED Inverted Laboratory Microscope to rule out cross-contamination with HeLa cells. The other cell lines were not authenticated.
Mycoplasma contamination	The cell lines were not tested for mycoplasma contamination.
Commonly misidentified lines (See ICLAC register)	See Authentication answer.

Animals and other research organisms

Policy information about studies involving animals; ARRIVE guidelines recommended for reporting animal research, and Sex and Gender in Research

Laboratory animals	Adult (7 to 8-week-old) C57BL/6J (000664) mice were obtained from the Jackson Laboratory (JAX).<br>A Cynomolgus macaque weighing 3.4 kg of age between 1.5 to 5 years was used by Charles River Laboratories.<br>Two rhesus macaques weighing 1 kg of age 3 months was used by the NIH Nonhuman Primate Testing Center for Evaluation of Somatic Cell Genome Editing Tools at the University of California, Davis.
Wild animals	No wild animals were used in the study.
Reporting on sex	All mice and the cynomolgus macaque used in this study were female. One rhesus macaque was female and the other was male.
Field-collected samples	No field collected samples were used in the study.
Ethics oversight	All mouse procedures were performed as approved by the Broad Institute Institutional Animal Care and Use Committee (IACUC).<br><br>For the cynomolgus macaque experiment, the study plan involving the care and use of animals was reviewed and approved by the Charles River CR-LAV Institutional Animal Care and Use Committee (IACUC). During the study, the care and use of animals was conducted by CR-LAV with guidance from the USA National Research Council and the Canadian Council on Animal Care (CCAC). The Test Facility is accredited by the CCAC and AAALAC. Per the CCAC guidelines, this study was considered as a category of invasiveness C.<br><br>The rhesus macaque study was conducted in the NIH Nonhuman Primate Testing Center for Evaluation of Somatic Cell Genome Editing Tools at the University of California, Davis. All procedures conformed to the requirements of the Animal Welfare Act, and protocols were approved prior to implementation by the UC Davis IACUC.

Note that full information on the approval of the study protocol must also be provided in the manuscript.
